# The immune correlates of protection for an avian influenza H5N1 vaccine in the ferret model using oil-in-water adjuvants

**DOI:** 10.1038/srep44727

**Published:** 2017-03-17

**Authors:** Sook-San Wong, Susu Duan, Jennifer DeBeauchamp, Mark Zanin, Lisa Kercher, Stephanie Sonnberg, Thomas Fabrizio, Trushar Jeevan, Jeri-Carol Crumpton, Christine Oshansky, Yilun Sun, Li Tang, Paul Thomas, Richard Webby

**Affiliations:** 1 Department of Infectious Diseases, St. Jude Children’s Research Hospital, Memphis, TN 38105, USA; 2 Department of Immunology, St. Jude Children’s Research Hospital, Memphis, TN 38105, USA; 3 Animal Resource Center, St. Jude Children’s Research Hospital, Memphis, TN 38105, USA; 4 Department of Biostatistics, St. Jude Children’s Research Hospital, Memphis, TN 38105, USA

## Abstract

Because of the pathogenicity and low incidence of avian influenza virus infections in humans, the immune correlates of protection for avian influenza vaccines cannot be determined from clinical studies. Here, we used the ferret model to address this for an avian influenza H5N1 vaccine. Using oil-in-water adjuvants, we generated groups of ferrets with undetectable (geometric mean titer [GMT] < 10), low (GMT = 28.3), or high (GMT > 761.1) hemagglutination-inhibition (HAI) titers to the A/Viet Nam/1203/2004 (H5N1) virus. Ferrets were then challenged with the wild-type virus and disease severity and immunologic parameters were studied. The severity of infection and symptom profile were inversely associated with pre-challenge HAI titers in a dose-dependent manner. A vaccinated ferret with no detectable HAI-antibodies but high flu-specific IgG-antibody titers mounted rapid functional antibodies after infection and experienced milder disease compared to other ferrets in the group. Compared to naïve ferrets, all vaccinated ferrets showed improved cellular immunity in the lungs and peripheral blood. High number of IFNγ^+^ CD8- T cells in the airways was associated with early viral clearance. Thus, while neutralizing antibodies are the best correlate of protection, non-neutralizing antibodies can also be protective. This should be taken into consideration in future avian influenza vaccine trials.

Viruses (AIV) are a significant threat to the human population, especially the H5N1 and H7N9 avian influenza viruses. To date, both viruses have caused over 1400 human cases with a 40–60% case fatality rate^1^,^2^ in laboratory-confirmed cases. As part of the pandemic preparedness plan, the United States Department of Health and Human Services has stockpiled vaccines against H5N1^3^ and H7N9 viruses through its Biomedical Advanced Research and Development Authority (BARDA). Testing the efficacies of these vaccines in available models is of considerable interest.

Early field studies with seasonal influenza vaccines showed that a hemagglutination-inhibition (HAI) titer greater than 40 is associated with protection from infection^4^, and this has often been used as the standard goal of immunogenicity in seasonal influenza vaccine studies. However, given the low prevalence of AIV infections in humans and the highly virulent nature of such infections, it is impossible to determine the immune correlate of protection from a vaccine trial. Thus, whether vaccination and the resulting immunity can protect against infection or disease caused by these viruses, is unknown. Studies in animal models have shown that HAI titers of <40 could mitigate the severity of infection (for examples, see refs 5, 6, 7), although none have examined the quantitative effects of antibody titers on infection outcome directly, or identified other immune factors that could mediate such protection.

We attempted to address this issue by using the ferret model. Ferrets are a standard animal model for studying influenza as they are able to reproduce the pathogenesis and contagiousness of the disease to a level that is relatively similar to that observed in humans^8^. We have previously shown in ferrets that pre-existing antibody titers, especially those of virus-neutralizing antibodies, correlate well with reduction in viral load in H7N9 infection^9^. However, the disease spectrum of H7N9 infection in ferrets is limited, and we were unable to assess the extent of vaccine protection in terms of disease severity. Compared with other avian influenza viruses, the H5N1 virus, particularly the A/Viet Nam/1203/2004 strain used in this study^1^, produces a pathogenic phenotype in ferrets^10^, enabling us to delineate the association of immune parameters with multiple aspects of disease severity.

We used adjuvants to induce different levels of HAI antibodies (the standard measure of inactivated influenza virus vaccine immunogenicity) in ferrets and subsequently challenged these ferrets with homologous wild-type virus to learn how different immunological factors might modulate the ensuing disease. The adjuvants used were two of the more successful squalene oil–in-water adjuvants that have been licensed in Europe, Canada, for select influenza vaccines in the US and were recently evaluated in trials with H7N9 and H5N1 vaccines^11^,^12^,^13^. Whilst H5N1 vaccine studies in ferrets are not novel, our adjuvanted vaccination regimen gave distinctive immunological profiles, which then enabled us to study the association between each immunological parameter within the context of subsequent infection. Thus, by using an animal model, this study helps bridge the gap left from vaccine trials by providing information on how vaccine-induced immunogenicity could potentially modulate the resulting disease severity.

## Results

**Figure 1:**
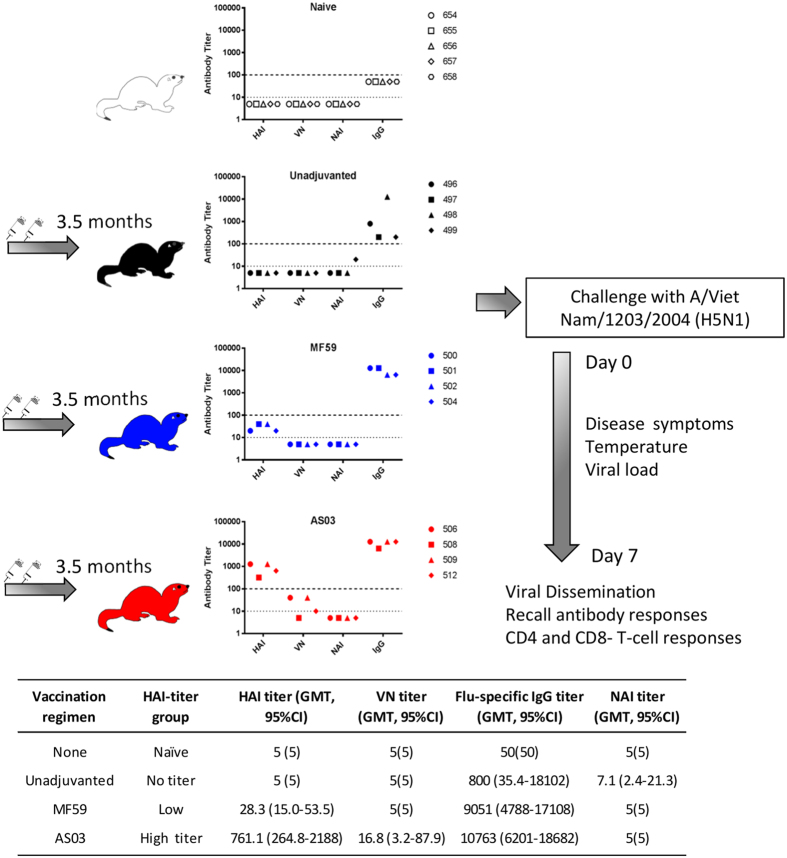
Schematic of experimental design and the antibody profile of each individual ferret prior to challenge. Ferrets were vaccinated with 2 doses of monovalent A/Viet Nam/1203/2004 (H5N1) (Sanofi-Pasteur) 3.5 months prior to challenge with homologous wild-type virus. Graph indicates the pre-challenge antibody titers measured at 3 months post-vaccination by hemagglutination-inhibition assay (HAI), virus neutralization (VN) assay, neuraminidase-inhibition (NAI) assay, and ELISA for vaccine-specific IgG-antibodies. Dashed line at Y = 10 indicates detection threshold for HAI, VN and NAI, while dashed line at Y = 100 indicates detection threshold for flu-specific IgG. Individual ferrets are assigned its respective symbol within its vaccine group (colored as ○ for naïve, ● for unadjuvanted, 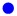 for MF59 and 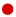 for AS03) and is tracked as such for the remainder of this manuscript. Summary statistics for the antibody titers (expressed as Geometric Mean Titers, GMT with 95% Confidence Intervals) are as listed in the summary table.

Three groups of ferrets were vaccinated with two doses of monovalent H5N1 vaccine (3.75 μg per dose) produced by Sanofi-Pasteur. One group received vaccine alone and two groups received vaccine in combination with either MF59 or AS03 adjuvants. Vaccinated ferrets (along with a group of age-matched unvaccinated ferrets) were bled at 3 months after receiving the last dose of vaccine to allow the antibody titers to stabilize and then challenged with 10^6^ EID_50_ of wild-type A/Viet Nam/1203/2004 (H5N1) 10 days later. The experimental design and the pre-challenge antibody titers to hemagglutinin (HA) and neuraminidase (NA) protein at 3 months post-vaccination are shown in Fig. 1. Our vaccination strategy resulted in three groups of ferrets with distinctly different pre-challenge HAI titers, categorized as undetectable (geometric mean titer [GMT] < 10), low (GMT = 28.3), and high (GMT = 761.1) (summarized in table in Fig. 1) that segregated with adjuvant type. Despite virus neutralization (VN) assays being more sensitive, we found that the VN titers were lower than HAI titers, especially in the AS03-group. These titers were similar even when tested with horse red blood cells, which were supposed to increase the assay sensitivity for avian influenza viruses^14^. One ferret in the undjuvanted group showed a neuraminidase-inhibition (NAI) titer of 20 prior to challenge (Fig. 1).

### Extent of protection correlated with pre-challenge HAI titer but IgG antibodies could reduce the severity of ensuing disease

Upon challenge, we assessed the severity of infection on the basis of weight loss, disease symptoms manifested, viral burden, and extrapulmonary virus replication. Of note, one ferret in the unadjuvanted group (499♦) succumbed to infection and we were not able to recover the tissue samples for subsequent *in vitro* assays.

**Figure 2:**
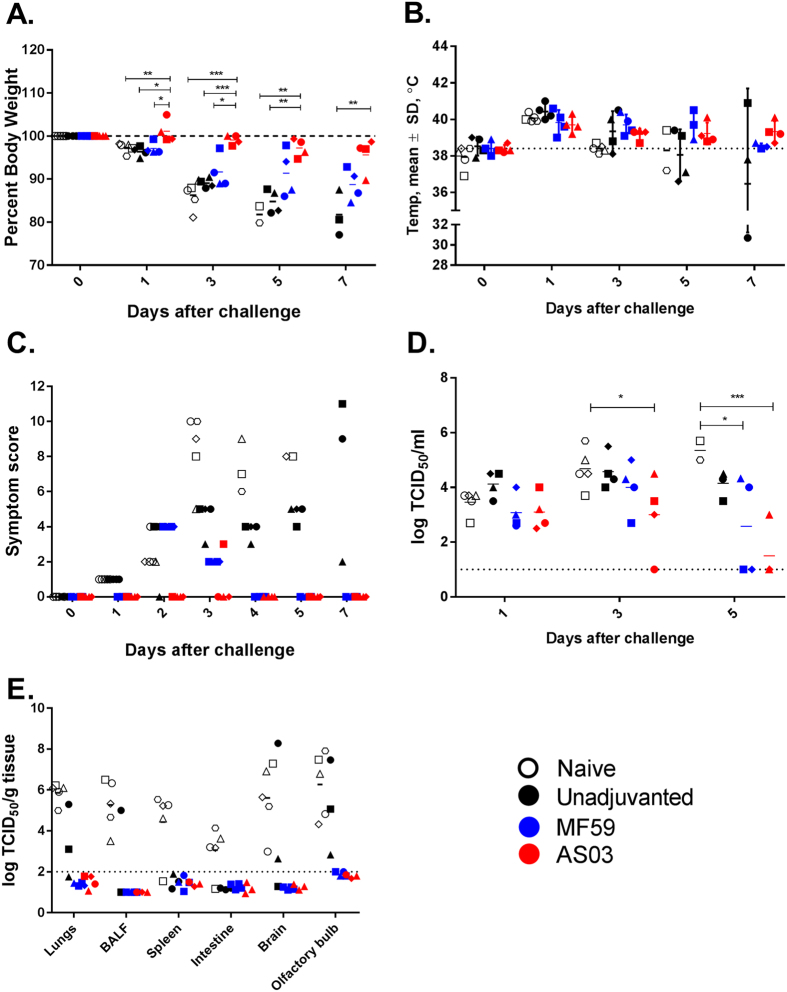
Disease severity in ferrets when challenged with wild-type A/Viet Nam/1203/2004 (H5N1). (**A**) Percent body weight. (**B**) Temperature change. (**C**) Cumulative symptom scores. (**D**) Viral load in the nasal cavity. (**E**) Replication of virus in extrapulmonary tissues after infection. Ferret 498 is indicated by ▲. *p < 0.05, **p < 0.01, and ***p < 0.001 by one-way ANOVA.

There appeared to be a close association between severity of infection and pre-challenge antibody titers. As expected, unvaccinated control ferrets succumbed rapidly to infection, and all five control ferrets were euthanized upon reaching the humane endpoints specified in the institutional protocol within 5 days. Ferrets in this group exhibited rapid weight loss and the most severe symptoms, which included extreme lethargy, inappetence, dehydration, and neurological symptoms (ataxia, hind-limb paresis, confusion, and, eventually, paralysis) (Fig. 2A–C and Table S1). They also had the highest viral burden, shedding high titers of virus in the upper respiratory tract (URT) and with widespread extrapulmonary virus replication (Fig. 2D,E). Ferrets in the unadjuvanted group, which had no pre-challenge HAI titer but some level of flu-specific IgG antibodies, fared only slightly better than the unvaccinated ferrets; they exhibited almost comparable weight loss but no or slower progression of severe symptoms (specifically, neurological-associated symptoms). Viral load in the nasal washes was lower in the unadjuvanted- group than in the naïve, unvaccinated group, but the difference was not statistically significant. However, extrapulmonary replication was restricted only to the brain in this group. Interestingly, ferret 498 (indicated by ▲), which had the highest pre-challenge flu-specific IgG titer in the unadjuvanted vaccine group, lost the least weight, was the least symptomatic, and had no viral replication in its lungs and only limited replication in its brain and olfactory bulb (Fig. 2A,C–E). Ferrets in the MF59-adjuvanted group, which had low HAI titers, showed less severe symptoms than the unadjuvanted group, i.e., only moderate weight loss, early resolution of symptoms, and viral replication restricted to the URT. In contrast, ferrets in the AS03-adjuvanted group, which had the highest pre-challenge HAI titers, were protected from severe disease. Ferrets in this group lost significantly less weight than those in the other groups, exhibited no severe symptoms, and had significantly less virus in the URT compared to unvaccinated ferrets, with viral shedding persisting for no more than 5 days. Replication was also restricted to the URT in this group. Interestingly, ferrets in the adjuvanted vaccine groups sustained higher body temperatures for longer than those in the unvaccinated or unadjuvanted vaccine groups, with temperatures in the unvaccinated ferrets declining rapidly after day 1 post-challenge.

Taken together, pre-challenge HAI titers did not prevent infection at this challenge dose but did modulate the subsequent disease profile in a dose-dependent manner. High pre-challenge HAI titers were associated with fever and, in the one ferret that showed mild symptoms, lethargy and inappetance after challenge. Ferrets with moderate HAI titers (or high IgG titers) showed fever, sustained lethargy and gastrointestinal symptoms. Absence of HAI or IgG antibodies was associated with neurological symptoms and rapid decline and eventually, death.

### Vaccinated ferrets showed improved cellular immune responses upon challenge compared to naïve ferrets

To examine the recall response, we evaluated the magnitude of the antibody and cellular immune responses in the bronchoalveolar lavage fluid (BALF) and blood at time of euthanasia or on day 7 post-challenge. The staining and gating strategy is provided as Supplemental Figure 1. CD8^+^ T-cells were identified by gating on the CD8^+^ population with the highest mean fluorescence intensity (MFI) as this population had been shown to be the true CD8^+^ T-cell population^15^. In gating for the CD4^+^ T-cells, we had to account for the unexpectedly high background fluorescence of the IFNγ-PE and CD8-APC into the FITC-channel (see Supplemental Figure 1). It was therefore necessary to set the CD4^+^ gate as such to avoid excessive background signal. We further normalized the residual background by subtracting the average signal from the -APC and –PE single stain controls from the CD4^+^-FITC signal.

**Figure 3:**
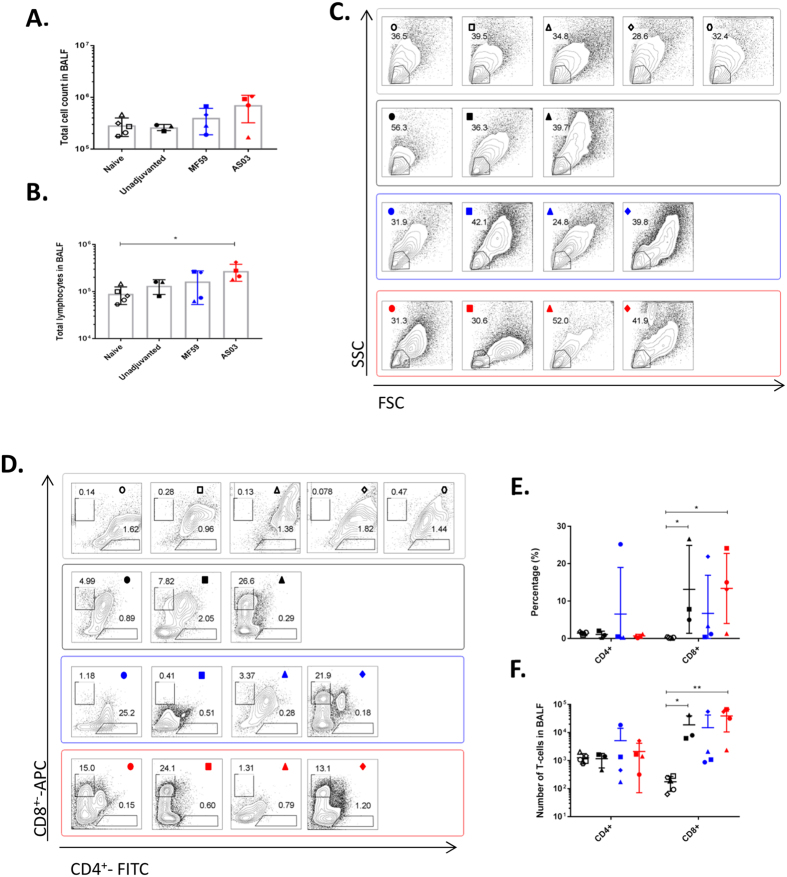
T-cell responses in the bronchoalveolar lavage fluid (BALF) at time of euthanasia or on day 7 post-challenge with wild-type A/Viet Nam/1203/2004 (H5N1). (**A**) Total cell infiltrate in the BALF. (**B**) Total number of lymphocytes in the BALF. (**C**) Flow plots showing the lymphocyte gates with the population frequency as indicated for each ferret, described in Fig. 1. This value was used to derive the absolute numbers in (**B**). (**D**) Flow plots to identify CD4^+^ and CD8^+^ T-cells in the BALF, with the respective frequencies as indicated. **(E)** Percentages and **(F)** absolute cell numbers of CD4^+^ and CD8^+^ T-cells in the BALF. All data are presented as group means and error bars represent ± standard deviation. * p < 0.05, ** p < 0.01 by Kruskal-Wallis test, followed by Dunn’s multiple comparison tests. Individual ferrets are assigned its respective symbol within its vaccine group (colored as ○ for naïve, ● for unadjuvanted, 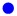 for MF59 and 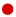 for AS03).

In the BALF, the numbers of infiltrating cells were generally higher in the MF59 and AS03 groups than the naïve and unadjuvanted groups although this difference did not reach statistical significance (Fig. 3A). The absolute number of infiltrating lymphocytes were found to be significantly higher in the AS03-group compared to the naïve group (p < 0.05) (Fig. 3B). All of the vaccinated animals had high numbers of infiltrating CD8^+^ T-cells compared to the naïve animals, while very minimal CD4^+^ T-cells were detected in most ferrets (Fig. 3D–F).

**Figure 4:**
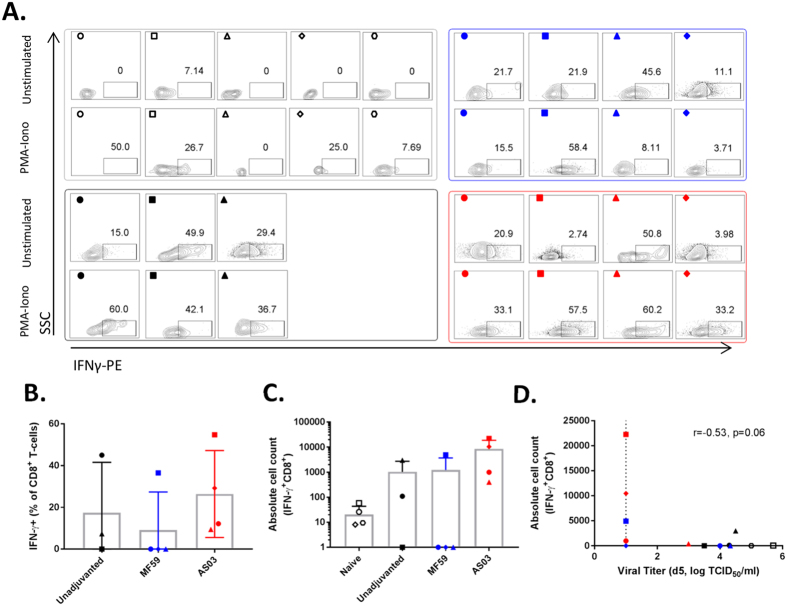
Levels of activated CD8^+^ T-cells in the bronchoalveolar lavage fluid (BALF). **(A)** Flow plots showing the percentage of IFN-γ^+^ cells within the CD8^+^ population for unstimulated and PMA/Ionomycin stimulated cells for each ferret as indicated in Fig. 1. The percentage in unstimulated were subtracted from the PMA/Ionomycin stimulated cells for each individual ferret to obtain the values shown in (**B**) and (**C**). Negative values are set at 0. **(B)** Normalized percentage and **(C)** absolute cell numbers of CD8^+^ IFN-γ^+^ T-cells after *ex vivo* stimulation of cells with the PMA/ionomycin mitogen cocktail. All data are presented as group means and error bars represent ±standard deviation. Correlation between **(D)** the absolute numbers of CD8^+^ IFN-γ^+^ T-cells with day 5 viral titers in the nasal wash. Correlation is determined using Pearson’s correlation coefficient, (r) with the p-value as indicated. Dashed line indicates limit of detection (LOD) of the virus titration assay. Ferrets with undetectable (negative) CD8^+^ IFN-γ^+^ activity were arbitrarily set at 1 cells/ml. Individual ferrets are assigned its respective symbol within its vaccine group (colored as ○ for naïve, ● for unadjuvanted, 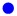 for MF59 and 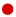 for AS03).

Due to the minimal detection of CD4^+^ T-cells, we concentrated on the IFNγ^+^-activity of the CD8^+^ T-cell population. After subtracting the unstimulated cells response from the PMA-Ionomycin stimulated response, the percentage of IFNγ^+^CD8^+^ T-cells were found to vary considerably within the groups (the naïve group was not included in Fig. 4B as the low CD8^+^ T-cells number resulted in inflated IFNγ^+^CD8^+^ T-cells percentages). Three ferrets in the MF59 group and ferret 497 (■) had high IFNγ^+^-activity in the unstimulated cells, resulting in no IFNγ^+^-activity after subtraction (Fig. 4A,B). However, in the remaining vaccinated ferrets, the absolute cell counts of IFNγ^+^CD8^+^ T-cells were 1- to 2- logs higher than in the naïve ferrets (Fig. 4C). Because CD8^+^ T-cells have been associated with rapid viral clearance in murine studies (reviewed in refs 16 and 17), we asked if this was also true in the ferrets. To ensure sufficient data points, we used viral titers in the nasal washes collected on day 5 as a measure of early viral clearance. The absolute numbers of IFNγ^+^CD8^+^ T-cells negatively correlated with viral titers on day 5 (Fig. 4D) (with statistical support of p = 0.06), suggesting that high numbers of infiltrating IFNγ^+^CD8^+^ T-cells was associated with earlier viral clearance.

**Figure 5:**
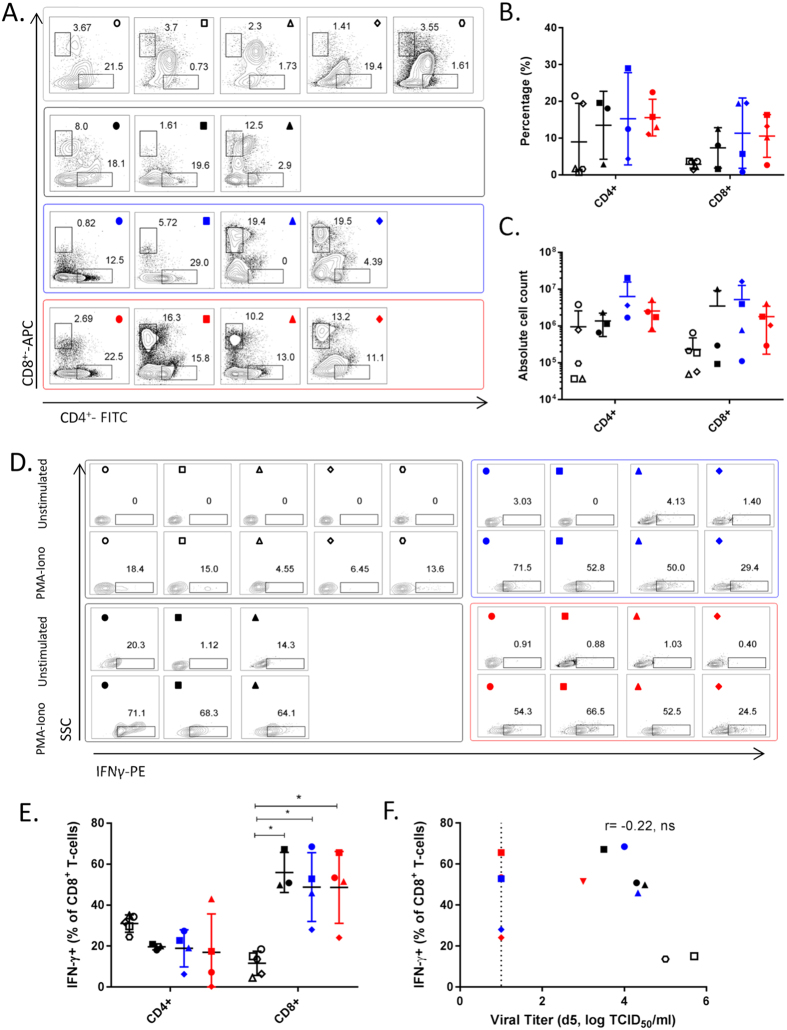
T-cell responses in the blood at time of euthanasia or on day 7 post-challenge with wild-type A/Viet Nam/1203/2004 (H5N1). (**A**) Flow plots to identify CD4^+^ and CD8^+^ T-cells in the blood for each ferret as indicated in Fig. 1. (**B**) Percentage and **(C)** absolute numbers of of CD4^+^ and CD8^+^ T-cells in the blood. (**D**) Flow plots showing the percentage of IFN-γ^+^ cells within the CD8^+^ population for unstimulated and PMA/Ionomycin stimulated cells for each ferret as indicated in Fig. 1. The percentage of unstimulated cells was subtracted from the PMA/Ionomycin stimulated cells for each individual ferret to obtain the values shown in (**E**). Negative values are set at 0. **(E)** Percentage of CD8^+^ IFN-γ^+^ T cells after *ex vivo* stimulation of PBMCs with the PMA/ionomycin mitogen cocktail. All data are presented as group means and error bars represent ±standard deviation. *p < 0.05, **p < 0.01 by Kruskal-Wallis test, followed by Dunn’s multiple comparison tests. **(F)** Correlation between the percentage of CD8^+^ IFN-γ^+^ T-cells with day 5 viral titers in the nasal wash. Correlation was determined using Pearson’s correlation coefficient, (r) with the p-value as indicated. Dashed line indicates limit of detection (LOD) of the virus titration assay. Individual ferrets are assigned its respective symbol within its vaccine group (colored as ○ for naïve, ● for unadjuvanted, 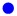 for MF59 and 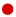 for AS03).

In the peripheral blood, there was a more discernable CD4^+^ T-cell population in most ferrets compared to that in the BALF (Fig. 5A), although some variability still existed. There was a trend of increased CD8^+^ T-cell numbers in some vaccinated ferrets compared to naïve ferrets (Fig. 5B,C) but this difference was not statistically significant. IFNγ^+^CD8^+^ T-cells levels were significantly higher in all the vaccinated ferrets compared to naïve ferrets (p < 0.05) (Fig. 5E). In contrast to the BALF, the IFNγ^+^CD8^+^ T-cell numbers in the blood did not correlate with early viral clearance (Fig. 5F).

In addition, there were no significant correlations between the T-cell populations in the BALF or blood with pre-challenge antibody titers (data not shown). We also noted a population of cells that were double-positive for both the CD4^+^ and CD8^+^ T-cells markers, particularly in the very sick ferrets. Whether these are artefacts or an unidentified population is currently unknown.

### Recall antibody response after challenge

**Figure 6:**
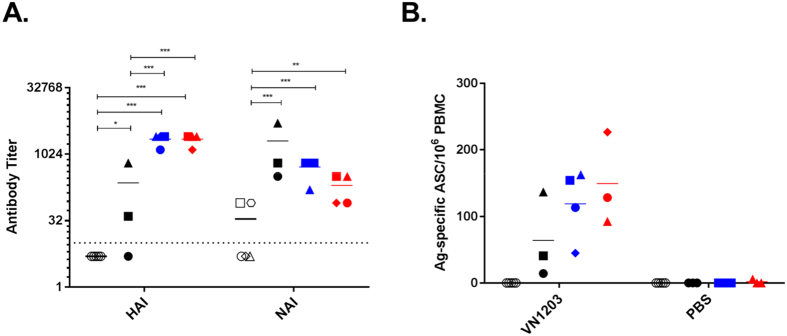
Antibody responses at the time of euthanasia or on day 7 post-challenge with wild-type A/Viet Nam/1203/2004 (H5N1). (**A**) Hemagglutinin (HA) and neuramidase (NA)-inhibiting-antibody titers as assayed by hemagglutination-inhibition (HAI) assay and enzyme-linked lectin assay (ELLA). (**B**) Number of H5-specific antibody-secreting cells in the peripheral blood as assayed by B-cell ELISPOT. Of note, we did not recover sufficient cells for one ferret in the AS03 group, and thus only 3 data points were available for this group. *p < 0.05, **p < 0.01, and ***p < 0.001 by one-way ANOVA. Individual ferrets are assigned its respective symbol within its vaccine group (colored as ○ for naïve, ● for unadjuvanted, 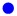 for MF59 and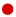 for AS03).

Upon challenge, the adjuvanted vaccine groups had comparable HAI titers that were significantly higher (GMT, MF59 = 2153, AS03 = 2153) than the unadjuvanted vaccine (50.4) and naïve (5) groups (p < 0.01, Fig. 6A). Functional NAI antibodies were detected within 5 days of infection in naïve ferrets but these titers (15.16) were significantly lower than those in vaccinated ferrets (p < 0.01). This suggests that, despite the lack of detectable pre-challenge NAI titers, vaccination induced some memory response against NA. Interestingly, there was a trend of higher NAI antibody titers in the unadjuvanted vaccine group (GMT = 1016) compared to the adjuvanted vaccine groups (MF59 = 452, AS03 = 160), although this difference was not statistically significant. The robust antibody response was also reflected in the elevated level of H5-specific IgG-secreting plasmablasts (Fig. 6B). Although the AS03 group had the highest number of antibody-secreting cells, the difference amongst vaccinated groups was not significant.

It is notable that ferret 498 in the unadjuvanted group, which had the highest pre-challenge vaccine-specific IgG titer and fared the best after infection (Table S1), showed an immune profile that, in some respects, was similar to ferrets with detectable HAI-titers. The BALF of this ferret had a high proportion of infiltrating lymphocytes, which also included IFNγ^+−^producing CD8^+^ T-cells (Figs 3 and 4). Ferret 498 also had the highest post-challenge HAI and NAI titers (640 and 5120, respectively), as well as high numbers of circulating antibody-secreting cells in its group. This suggests that, functional antibodies could be rapidly induced upon recall, despite its’ early absence (Fig. 6B).

In summary, high pre-challenge antibody titers were associated with protection after infection and were associated with good recall immunologic response. Protection was best when functional/neutralizing (HAI) antibodies were induced, which appeared to prevent viral dissemination beyond the URT. Vaccinated ferrets that were better protected demonstrated a more pronounced CD8^+^ T-cell response, compared to those that were severely sick.

### Association of pre-challenge titers with disease severity

**Figure 7:**
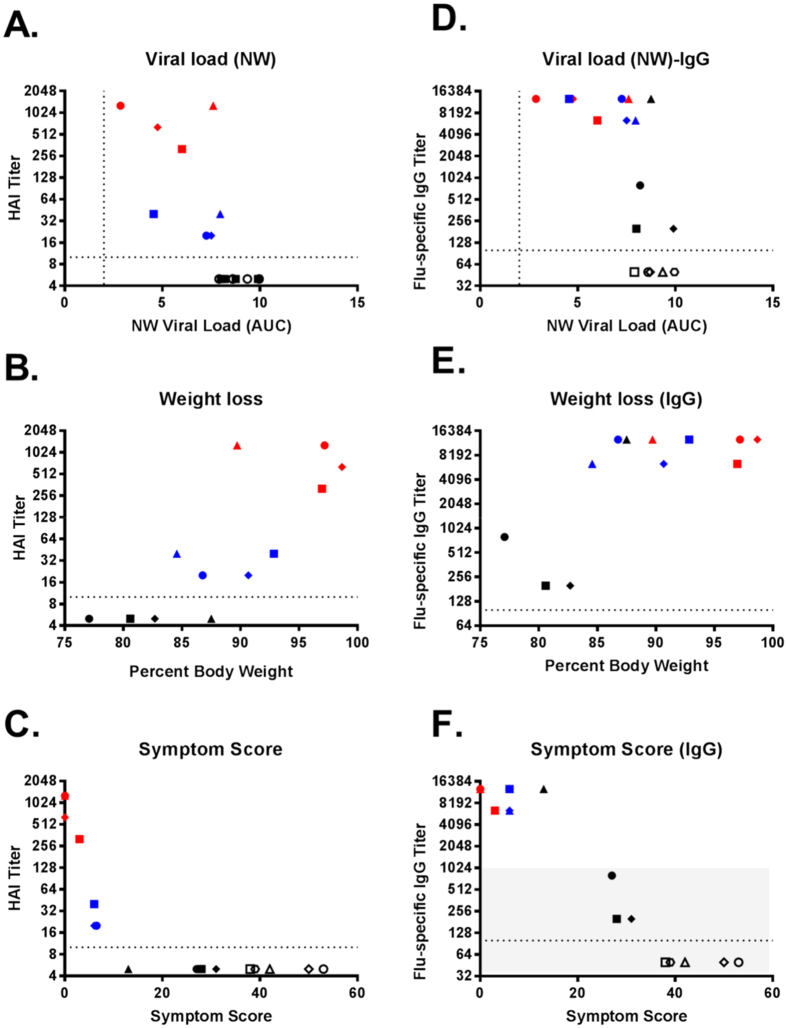
Relationship between pre-challenge HAI or IgG titers and measured clinical parameters after infection. Relationship between pre-challenge HAI or vaccine-specific IgG antibodies with (**A,D**) cumulative viral load in the nasal washes (NW), (**B,E**) weight loss and (**C,F**) cumulative symptom score. The total viral load were derived from Area-Under-the Curve (AUC) calculations in Fig. 2D. Cumulative symptom scores were tabulated in Table S1 over the 7 study-days. Ferrets that were euthanized were allocated the maximum score of 13 on days indicated with an **E** in Table S1. The area shaded in gray in (**F**) indicates ferrets that were euthanized. Individual ferrets are assigned its respective symbol within its vaccine group (colored as ○ for naïve, ● for unadjuvanted, 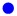 for MF59 and 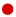 for AS03).

To obtain a measure of protection, we plotted the pre-challenge HAI and IgG titers against the different clinical parameters measured after challenge in each individual ferret (Fig. 7). Overall, the trend of dose-dependent protection was less striking for the ferrets within each vaccine group than amongst the group. This is because the differences in antibody titers were smaller within the group compared to the differences observed amongst the groups. HAI antibody titers correlated better with reduction in overall viral load in the nasal cavity than did IgG-antibodies (Fig. 7A,D). IgG-antibody titers, however, correlated better with symptom scores, although ferrets with IgG-titers of less than 10^3^ logs did not survive the challenge (Fig. 7C,F). As before, ferret 498 was protected to a greater degree than the other ferrets in that group.

## Discussion

H5N1 vaccine studies in ferrets are not novel, but detailed studies on the association of antibody levels with the outcome of infection are few. Not unexpectedly, HAI antibodies remained the best correlate of protection, even though they did not provide sterilizing immunity at this study’s challenge dose. We showed here that even low HAI titers could still reduce disease severity-resulting in a moderate symptom profile in the ferrets (i.e diarrhea). Any detectable HAI antibodies (by the limits of current assays) appeared to prevent neurological involvement- something that even high IgG-titers were not sufficient for (as summarized in Table 1). However, as seen in ours’ and others’ studies, the absence of an HAI titer does not necessarily preclude protection: vaccinated ferrets appear to be partially protected despite the absence of HAI antibodies when challenged with H5N1 or H7N9 virus^7^,^18^,^19^. We show here that this protection is mediated by the induction of cellular immunity and facilitated by high titers of pre-existing influenza-specific IgG-antibodies.

All vaccinated ferrets showed improved cellular immunity compared to naïve ferrets at the site of infection and in the peripheral blood. Notably, the role for CD8^+^ T cells in restricting virus replication in the airways, which has only been shown in murine studies (reviewed in refs 16 and 17), was also evident in our study. In addition, high pre-challenge virus-specific IgG antibody titer was associated with improved cellular immunity, as exemplified by ferret 498. When challenged, this ferret had robust CD8^+^ T-cells and recall antibody response and a symptom profile almost comparable to those of ferrets with pre-challenge HAI titers. Several studies evaluating heterosubtypic influenza immunity have demonstrated that T-cell mediated protection is most effective when there is sufficient engagement of B-cells, and that non-neutralizing antibodies can improve virus clearance and reduce pathology through Fc-dependent phagocytosis or antibody-dependent-cellular cytotoxicity (ADCC) processes^20^,^21^,^22^,^23^. Since inactivated vaccines are generally acknowledged to be poor inducers of T-cell memory responses, it is likely that non-neutralizing antibodies generated by the vaccine cooperated with CD8^+^ memory or effector T cells after infection to induce a robust T-cell response. This cooperative requirement for optimal protection could explain why the remaining ferrets in the unadjuvanted vaccine group that had low IgG-titers were not protected to the same capacity as ferret 498. Thus, while circulating HAI antibodies were needed to more effectively limit virus replication and spread, pre-existing IgG antibodies can reduce disease severity by engaging T-cell immunity.

Another notable observation was the levels of recall antibodies to NA among the ferret groups. Despite the lack of detectable pre-challenge NAI titers, all vaccinated ferrets produced significantly more NAI antibodies than naïve ferrets. NAI antibody levels in vaccinated ferrets were inversely associated with pre-challenge HAI antibody levels, being lowest in the AS03-adjuvanted vaccine group and highest in the unadjuvanted vaccine group. We have previously determined that this particular lot of vaccine contained very little NA (data not shown), thus the NA-specific memory B cells were probably only minimally induced. Since we and others have shown that antigen concentration could bias the specificity of the resulting antibody response after adjuvanted vaccine administration^9^,^24^, this trend suggests that the B-cell memory pool induced by the adjuvanted vaccine is HA-dominant and result in a recall antibody response that is likely biased towards HA.

There were several limitations to the present study. The major limitation was, despite our best efforts, the CD4^+^ T-cell populations were not well-defined in our flow analyses. We did not detect any significant CD4^+^ T-cell numbers in the BALF and the numbers were highly varied in the peripheral blood, particularly for the ferrets in the naïve group with more severe disease. A study published recently^15^ reported an average of 40% CD4^+^ and 15% CD8^+^ T-cells in the BALF of two ferrets infected with the H1N1 virus at day 10 post-infection. Whether the early time-point and the rapidly declining health of our ferrets, or a suboptimal detection antibody, confounded our ability to reliably detect the CD4^+^ T-cells, is uncertain. The role of CD4^+^ T-cells in mediating protection against H5N1 virus infection should therefore be evaluated in future studies with better reagents.

Another limitation was that our experimental design did not allow for detailed study of the kinetics of immune response and viral clearance. A difference in response kinetics could explain why we detected no differences in the magnitude of response among the vaccinated groups^25^,^26^. This limitation could be overcome in future studies with a more detailed sampling protocol to address the immune kinetics of the adjuvanted versus nonadjuvanted vaccine-induced T-cell response.

Finally, although the ferret model is the best mammalian model to replicate the pathogenesis of highly pathogenic H5N1 infection, it still may not accurately reflect the complexity of the disease progression in humans. H5N1 infections in humans cause acute respiratory distress syndrome and death is often due to respiratory failure with multiorgan involvement. In reported cases, viremia, gastrointestinal symptoms and extrapulmonary spread is not uncommon in severe H5N1 cases, but central nervous system (CNS) involvement is rare^27^,^28^. In the ferret model, the typical challenge approach such as that used in this study often results in CNS infection via the olfactory nerve^29^,^30^. While we are not able to extrapolate the ability of neutralizing antibodies in preventing CNS involvement in humans, our data does indicate that the presence of neutralizing antibodies prevents systemic virus spread. In addition, the challenge dose we used was high. It is possible that with a lower infection dose, the immune requirement for protection would be less.

MF59 and AS03 represent two of the more successful adjuvants in the market, and have improved the immunogenicity of avian influenza vaccines considerably in recent vaccine trials. Both are squalene-based emulsions that act by stimulating a local inflammatory environment, although AS03 has an added component of α-tocopherol (Vitamin E), an immunostimulant^31^ that was important for its potent adjuvant effect^32^. However, this difference in potency is negligible when antigen concentration is increased^9^. Certainly, Focetria™ and Pandemrix™ (MF59- and AS03- adjuvanted monovalent 2009 pandemic H1N1 vaccine, which are licensed for use in Europe), are formulated in 7.5 μg and 3.75 μg dose respectively. Recent human trials of H5N1 vaccines with or without MF59 adjuvant at two doses of 7.5 μg were able to induce an HAI GMT of 63 in approximately 83% of participants^12^. Similarly, two doses of AS03-adjuvanted H5N1 vaccine induced a GMT of more than 100 in 100% of vaccinees^11^. These adjuvanted vaccine formulations (albeit with contemporary H5N1 strains) represent a considerable improvement over the unadjuvanted formulation or those used in earlier trials with a high HA dose^33^. Furthermore, cross-reactive antibodies against variant strains can also be induced with adjuvanted vaccines^11^,^19^. In the absence of a viable experimental setting to determine the immune correlate of protection for the H5N1 vaccine, our data suggests that any H5N1 vaccine regimen that is capable of inducing HAI-antibodies would be protective. Although we showed that high pre-existing HAI titer is ideal for the prevention of severe symptoms, functional antibodies can still be induced rapidly upon infection and confer moderate protection with low pre-existing HAI titers. Even in the absence of HAI antibodies, vaccination can induce levels of virus-specific IgG and T-cell immunity sufficient to delay disease progression. This is a critical consideration for timely antiviral treatments or other forms of intervention, as delayed onset of treatment is often associated with a fatal outcome^34^,^35^. Assays to evaluate non-neutralizing antibody levels as an additional parameter should thus be considered in future vaccine trials.

## Methods

### Vaccine and adjuvants

The monovalent A/Viet Nam/1203/2004 (H5N1) (Sanofi-Pasteur) vaccine was a commercially manufactured split-virion vaccine derived from the reverse-genetics attenuated virus strain^36^. MF59 is owned by Seqirus, CSL (formerly by Novartis) and AS03 by GlaxoSmithKline. All vaccines and adjuvants were received through the Office of Biomedical Advanced Research and Development Authority (BARDA).

### Animals

Specific-pathogen–free ferrets, 4 to 6 months old, were purchased from Triple F Farms (Bradford County, PA). All animal experiments were approved and performed according to guidelines set by the St. Jude Children’s Research Hospital’s Animal Care and Use Committee in an enhanced animal biosafety level 3+ containment facility. All animals were determined to be seronegative by HAI to circulating seasonal influenza strains prior to the start of the experiment.

### Viruses and cells

The attenuated virus strains used in subsequent serological assays were generated with the HA and NA from the virus of interest (A/Viet Nam/1203/2004 [H5N1]), with the polybasic cleavage site in the HA removed, and the six internal genes from A/Puerto Rico/8/1934 (PR8).

Virus stocks were prepared by propagation in 10-day-old embryonated chicken eggs at 35 °C for 36 hours, aliquoted, and stored at −70 °C. Marin-Darby canine kidney (MDCK) cells used for virus titration were propagated in minimal essential medium (MEM) supplemented with 10% fetal calf serum, vitamins, L-glutamine, and antibiotics at 37 °C in a humidified 5% CO_2_ environment.

### Immunization dose and schedule

Briefly, three groups of ferrets (four per group) received two doses of H5N1 vaccine (3.75 μg HA/dose) administered 3 weeks apart in 0.75 mL volume by intramuscular injection. Vaccine was diluted in saline to achieve the desired dose and mixed with either MF59 or AS03 in a 1:1 ratio. All adjuvanted vaccine groups received the same amount of adjuvant. Pre-challenge sera were collected at 100 days after administration of the second vaccine dose to allow the antibody titers to stabilize and to avoid non-specific effects due to the adjuvants.

### Virus challenge and tissue sampling

The challenge experiment was performed 110 days after the second dose of vaccine was administered. Five naïve, unvaccinated ferrets were included as controls for the challenge. Ferrets were anesthetized with isoflurane and inoculated intranasally with 1 mL of 10^6^ EID_50_ of wild-type H5N1 A/Viet Nam/1203/2004 virus. Clinical signs of infection, weight loss, and temperature were monitored daily up to 7 days post challenge.

On days 3, 5, and 7 after virus inoculation, ferrets were anesthetized with ketamine, and nasal washes were collected in 1 mL of phosphate-buffered saline (PBS). Ferrets were euthanized at day 7 or upon meeting the humane endpoints specified in the institutional protocol. Tissue specimens were collected from the lungs (pooled from each lobe), spleen, small and large intestine (0.5 cm of each section, pooled), brain, and olfactory bulb. Tissues were homogenized, titrated on MDCK cells and the virus titer was determined by using the Reed and Muench method^37^. The limit of virus detection was less than 1 log_10_ TCID_50_/mL.

For immunological assays, whole blood was collected in BD Vacutainer^®^ CPT™ tubes supplemented with sodium heparin (BD Biosciences), and peripheral blood mononuclear cells (PBMCs) were isolated according to the manufacturer’s protocol. Bronchoalveolar lavage fluid (BALF) was collected by flushing the lungs with 25 mL of PBS via an endotracheal tube (Rusch). Supernatant collected from the BALF was also used in virus titration experiments.

### B-cell enzyme-linked immunospot (ELISPOT) assay

Isolated PBMCs were plated at 3 × 10^5^ cells/well in wells coated with 2000 HA units/mL of BPL-inactivated rg (6 + 2) A/Viet Nam/1203/2004 (H5N1). The next day, cells were removed and the plate was washed with PBS and PBS containing 0.05% Tween-20 (PBS-T) (Sigma). The plate was then incubated with peroxidase-conjugated anti–ferret IgG-HRP (Novus Biologicals) and finally developed with 3,3′,5,5′-tetramethylbenzidine (TMB) substrate.

### *In vitro* stimulation and flow cytometry

PBMCs were plated at 1 × 10^5^ cells/well in a 96-well plate. To each well were added either 40 ng/ml phorbol-myristate-acetate (PMA), and 500 ng/ml ionomycin cocktail or media alone. After overnight incubation at 37 °C, cells were treated with Brefeldin A (eBioscience) for 4 h. Cell-surface staining was performed with FITC-conjugated anti-ferret CD4 (Sino Biological) and rabbit-raised anti–ferret CD8 (Sino Biological). A secondary APC-conjugated anti-rabbit antibody (Molecular Probes) was added in a second incubation step. For intracellular IFNγ^+^ staining, cells were permeabilized with 0.5% saponin, which was followed by staining with PE-conjugated mouse-anti–bovine IFNγ antibody (AbD Serotec). Approximately 100,000 events were then acquired using a FACSCalibur flow cytometry system (BD Biosciences) and analyzed with FlowJo (v10) software. The general gating strategy is included in Supplemental Figure 1.

### Hemagglutinin-inhibition (HAI) and virus neutralization (VN) assays

Serum samples were treated with receptor-destroying enzyme (Denka Seiken), heat-inactivated at 56 °C for 30 min, and tested in an HAI assay with 0.5% chicken red blood cells^38^. A virus neutralization assay was performed according to World Health Organization (WHO) guidelines^39^. Neutralization titer was determined as the reciprocal dilution that inhibited virus growth in a hemagglutination assay.

### Enzyme-linked immunosorbent assay (ELISA)

Briefly, serum samples were serially diluted and tested for influenza-specific IgG titers as previously described^9^. 0.1 μg/mL of the vaccine was used as coating antigen.

### Enzyme-linked lectin assay (ELLA) for detection of neuraminidase antibodies

The presence of NA-specific antibodies was determined using the enzyme-linked lectin assay (ELLA)^39^ using a recombinant reverse-genetics virus containing the NA from A/Viet Nam/1203/2004 (H5N1), together with a mismatched HA from A/Teal/Hong Kong/W312/1997 (H6N2) as antigen as previously described^9^,^39^.

### Statistical tests

Data were analyzed using GraphPad Prism, version 5.03 and 7.0. Comparison of antibody responses was performed using log_10_-transformed antibody titers and analysis of variance (ANOVA) with Bonferroni’s corrections applied for multiple comparisons. Viral titers were expressed as mean titer ± standard deviation in log_10_ TCID_50_/mL (or TCID_50_/g for tissues). Weight loss, temperature, viral titers in nasal washes, and immunological data were compared by ANOVA. Flow cytometric data were analyzed using non-parametric methods. Correlation between T-cell responses with viral titers were analyzed using Pearson’s Correlation test. A *P*-value of 0.05 or less was considered statistically significant.

## Additional Information

**How to cite this article:** Wong, S.-S. *et al*. The immune correlates of protection for an avian influenza H5N1 vaccine in the ferret model using oil-in-water adjuvants. *Sci. Rep.* **7**, 44727; doi: 10.1038/srep44727 (2017).

**Publisher's note:** Springer Nature remains neutral with regard to jurisdictional claims in published maps and institutional affiliations.

## Supplementary Material

Supplementary Information

## Figures and Tables

**Table 1:** Extent of protection within each immunization group.

Vaccine Group	Pre-challenge			Post-challenge										
	Antibody response^**a**^			Clinical Manifestations				Viral Burden			Antibody response		Cellular Immunity	
	HAI	Flu-specific IgG	NAI	Weight Loss^b^	Fever > 5 days	Neurological symptoms^c^	Diarrhea^c^	Death^c^	Nasal cavity^d^	Extrapulmonary spread	HAI	NAI	Activated CD4 + /CD8 + in BALF	Activated CD4 + /CD8 + in blood
Naïve	n.d	n.d	n.d	+++	One	100%	80%	100%	High	Widespread	n.d	low	n.d	n.d
No Titer	n.d	+	Low	+++	Yes	25%	100%	25%	High	Brain/Olfactory Bulb	none-moderate	Moderate-High	CD4 < CD8	CD4 < CD8
Low Titer	Moderate	++	n.d	++	No	0%	100%	0%	50% ferrets cleared	None	High	Low-moderate	CD4 < CD8	CD4 < CD8
High Titer	High	+++	n.d	+	No	0%	25%	0%	Mostly cleared	None	High	Low-moderate	CD4 < CD8	CD4 < CD8

^a^GMT of antibody titer per group- n.d: not detected, +: 10^1^–10^2^, ++: 10^1^–10^2^, +++: 10^2^–10^3^.

^b^Percentage weight loss from starting weight: + = < 5%, + + = 5–10%, + + + = > 10%.

^c^Percentage of ferrets within group that exhibited these symptoms. Death is considered according to the institutional animal protocol.

^d^Based on viral titers recovered in nasal washes at day 5 post-challenge. High >10^4^ logs TCID_50_.
