# Supplementary material for: The immune correlates of protection for an avian influenza H5N1 vaccine in the ferret model using oil-in-water adjuvants
Source: Sci Rep. 2017 Mar 17;7:44727. doi: 10.1038/srep44727 (PMC5381113; doi:10.1038/srep44727)

Supplemental Material

**The immune correlate of protection for an avian influenza H5N1 vaccine in the ferret model by using oil-in-water adjuvants**

Sook-San Wong^1^, Susu Duan^2^, Jennifer DeBeauchamp^1^, Mark Zanin^1^, Lisa Kercher^3^, Stephanie Sonnberg^1,5^, Thomas Fabrizio^1^, Trushar Jeevan^1^, Jeri-Carol Crumpton^1^, Christine Oshansky^2,6^, Yilun Sun^4^, Li Tang^4^, Paul Thomas^2^, and Richard Webby^1^*

**Affiliations:**

^1^Department of Infectious Diseases, St. Jude Children’s Research Hospital, Memphis, TN 38105, USA.

^2^Department of Immunology, St. Jude Children’s Research Hospital, Memphis, TN 38105, USA.

^3^Animal Resource Center, St. Jude Children’s Research Hospital, Memphis, TN 38105, USA.

^4^Department of Biostatistics, St. Jude Children’s Research Hospital, Memphis, TN 38105, USA.

^5^Present affliation: Takeda Vaccines, 504 South Rosa Road suite 200, Madison, WI 53719, USA.

^6^ Present affliation: Biomedical Advanced Research and Development Authority (BARDA) Office of the Assistant Secretary for Preparedness and Response (ASPR), U. S. Department of Health and Human Services (DHHS),200 C Street, SW Washington, DC 2020, USA

*Corresponding author: Richard J. Webby

**Phone:** +1 (901) 595-2243.

**E-mail:** Richard.Webby@stjude.org

**Table S1.** Clinical scores for each ferret after challenge with wild-type A/Viet Nam/1203/2004 (H5N1). Lethargy was scored based on activity level as 0: alert and playful; 1: alert, but only playful when stimulated; 2: alert, but not playful even when stimulated; or 3: neither alert nor playful when stimulated. Neurological distress was scored based on severity as 1: slight; 2: severe; or 3: paralysis. Respiratory distress was scored as 1: sneezing; 2: discharge; or 3: labored breathing. E indicates an animal that was euthanized. Euthanized animals were assigned maximum pathology score of 13.

| **Day** | **Symptoms** | **Unadjuvanted** | | | | **MF59** | | | | **ASO3** | | | | **Unvaccinated** | | | | |
| --- | --- | --- | --- | --- | --- | --- | --- | --- | --- | --- | --- | --- | --- | --- | --- | --- | --- | --- |
|  |  | **496** | **497** | **498** | **499** | **500** | **501** | **502** | **504** | **506** | **508** | **509** | **512** | **654** | **655** | **656** | **657** | **658** |
| 1 | Lethargy | 0 | 0 | 0 | 0 | 0 | 0 | 0 | 0 | 0 | 0 | 0 | 0 | 0 | 1 | 1 | 1 | 1 |
|  | Inappetence | 0 | 0 | 0 | 0 | 0 | 0 | 0 | 0 | 0 | 0 | 0 | 0 | 0 | 0 | 0 | 0 | 0 |
|  | Dehydration | 0 | 0 | 0 | 0 | 0 | 0 | 0 | 0 | 0 | 0 | 0 | 0 | 1 | 0 | 0 | 0 | 0 |
|  | Neurological distress | 0 | 0 | 0 | 0 | 0 | 0 | 0 | 0 | 0 | 0 | 0 | 0 | 0 | 0 | 0 | 0 | 0 |
|  | Diarrhea | 1 | 1 | 1 | 1 | 0 | 0 | 0 | 0 | 0 | 0 | 0 | 0 | 0 | 0 | 0 | 0 | 0 |
|  | Vomiting | 0 | 0 | 0 | 0 | 0 | 0 | 0 | 0 | 0 | 0 | 0 | 0 | 0 | 0 | 0 | 0 | 0 |
|  | Respiratory distress | 0 | 0 | 0 | 0 | 0 | 0 | 0 | 0 | 0 | 0 | 0 | 0 | 0 | 0 | 0 | 0 | 0 |
|  | Total score | **1** | **1** | **1** | **1** | **0** | **0** | **0** | **0** | **0** | **0** | **0** | **0** | **1** | **1** | **1** | **1** | **1** |
| 2 | Lethargy | 1 | 1 | 1 | 1 | 1 | 1 | 1 | 1 | 0 | 1 | 0 | 0 | 2 | 2 | 1 | 1 | 2 |
|  | Inappetence | 1 | 1 | 1 | 1 | 1 | 1 | 1 | 1 | 0 | 1 | 0 | 0 | 1 | 0 | 0 | 0 | 0 |
|  | Dehydration | 1 | 1 | 1 | 1 | 1 | 1 | 1 | 1 | 0 | 1 | 0 | 0 | 1 | 0 | 1 | 1 | 0 |
|  | Neurological distress | 0 | 0 | 0 | 0 | 0 | 0 | 0 | 0 | 0 | 0 | 0 | 0 | 0 | 0 | 0 | 0 | 0 |
|  | Diarrhea | 1 | 1 | 0 | 1 | 1 | 1 | 1 | 1 | 0 | 0 | 0 | 0 | 0 | 0 | 0 | 0 | 0 |
|  | Vomiting | 0 | 0 | 0 | 0 | 0 | 0 | 0 | 0 | 0 | 0 | 0 | 0 | 0 | 0 | 0 | 0 | 0 |
|  | Respiratory distress | 0 | 0 | 0 | 0 | 0 | 0 | 0 | 0 | 0 | 0 | 0 | 0 | 0 | 0 | 0 | 0 | 0 |
|  | Total score | **4** | **4** | **3** | **4** | **4** | **4** | **4** | **4** | **0** | **3** | **0** | **0** | **4** | **2** | **2** | **2** | **2** |
| 3 | Lethargy | 2 | 2 | 1 | 2 | 1 | 1 | 1 | 1 | 0 | 1 | 0 | 0 | 3 | 3 | 2 | 3 | 3 |
|  | Inappetence | 1 | 1 | 1 | 1 | 1 | 1 | 1 | 1 | 0 | 1 | 0 | 0 | 1 | 1 | 1 | 1 | 1 |
|  | Dehydration | 1 | 1 | 1 | 1 | 0 | 0 | 0 | 0 | 0 | 1 | 0 | 0 | 1 | 1 | 1 | 1 | 1 |
|  | Neurological distress | 0 | 0 | 0 | 0 | 0 | 0 | 0 | 0 | 0 | 0 | 0 | 0 | 2 | 0 | 0 | 3 | 2 |
|  | Diarrhea | 1 | 1 | 0 | 1 | 0 | 0 | 0 | 0 | 0 | 0 | 0 | 0 | 0 | 0 | 1 | 1 | 0 |
|  | Vomiting | 0 | 0 | 0 | 0 | 0 | 0 | 0 | 0 | 0 | 0 | 0 | 0 | 0 | 0 | 0 | 0 | 0 |
|  | Respiratory distress | 0 | 0 | 0 | 0 | 0 | 0 | 0 | 0 | 0 | 0 | 0 | 0 | 3 | 3 | 0 | 0 | 3 |
|  | Total score | **5** | **5** | **3** | **5** | **2** | **2** | **2** | **2** | **0** | **3** | **0** | **0** | **10** | **8** | **5** | **9** | **10** |
| 4 | Lethargy | 2 | 2 | 2 | 2 | 0 | 0 | 0 | 0 | 0 | 0 | 0 | 0 | E | 3 | 3 | E | 2 |
|  | Inappetence | 1 | 1 | 1 | 1 | 0 | 0 | 0 | 0 | 0 | 0 | 0 | 0 | E | 1 | 1 | E | 1 |
|  | Dehydration | 1 | 1 | 0 | 1 | 0 | 0 | 0 | 0 | 0 | 0 | 0 | 0 | E | 1 | 1 | E | 1 |
|  | Neurological distress | 0 | 0 | 0 | 0 | 0 | 0 | 0 | 0 | 0 | 0 | 0 | 0 | E | 1 | 3 | E | 2 |
|  | Diarrhea | 0 | 0 | 0 | 0 | 0 | 0 | 0 | 0 | 0 | 0 | 0 | 0 | E | 1 | 1 | E | 0 |
|  | Vomiting | 0 | 0 | 0 | 0 | 0 | 0 | 0 | 0 | 0 | 0 | 0 | 0 | E | 0 | 0 | E | 0 |
|  | Respiratory distress | 0 | 0 | 0 | 0 | 0 | 0 | 0 | 0 | 0 | 0 | 0 | 0 | E | 0 | 0 | E | 0 |
|  | Total score | **4** | **4** | **3** | **4** | **0** | **0** | **0** | **0** | **0** | **0** | **0** | **0** | **13** | **7** | **9** | **13** | **6** |
| 5 | Lethargy | 3 | 2 | 3 | 3 | 0 | 0 | 0 | 0 | 0 | 0 | 0 | 0 | E | 3 | E | E | 2 |
|  | Inappetence | 1 | 1 | 1 | 1 | 0 | 0 | 0 | 0 | 0 | 0 | 0 | 0 | E | 1 | E | E | 1 |
|  | Dehydration | 1 | 1 | 1 | 1 | 0 | 0 | 0 | 0 | 0 | 0 | 0 | 0 | E | 1 | E | E | 1 |
|  | Neurological distress | 0 | 0 | 0 | 0 | 0 | 0 | 0 | 0 | 0 | 0 | 0 | 0 | E | 2 | E | E | 3 |
|  | Diarrhea | 0 | 0 | 0 | 0 | 0 | 0 | 0 | 0 | 0 | 0 | 0 | 0 | E | 1 | E | E | 1 |
|  | Vomiting | 0 | 0 | 0 | 0 | 0 | 0 | 0 | 0 | 0 | 0 | 0 | 0 | E | 0 | E | E | 0 |
|  | Respiratory distress | 0 | 0 | 0 | 3 | 0 | 0 | 0 | 0 | 0 | 0 | 0 | 0 | E | 0 | E | E | 0 |
|  | Total score | **5** | **4** | **5** | **8** | **0** | **0** | **0** | **0** | **0** | **0** | **0** | **0** | **13** | **8** | **13** | **13** | **8** |
| 7 | Lethargy | 3 | 3 | 2 | E | 0 | 0 | 0 | 0 | 0 | 0 | 0 | 0 |  |  |  |  |  |
|  | Inappetence | 1 | 1 | 0 | E | 0 | 0 | 0 | 0 | 0 | 0 | 0 | 0 |  |  |  |  |  |
|  | Dehydration | 1 | 1 | 0 | E | 0 | 0 | 0 | 0 | 0 | 0 | 0 | 0 |  |  |  |  |  |
|  | Neurological Distress | 0 | 2 | 0 | E | 0 | 0 | 0 | 0 | 0 | 0 | 0 | 0 |  |  |  |  |  |
|  | Diarrhea | 0 | 0 | 0 | E | 0 | 0 | 0 | 0 | 0 | 0 | 0 | 0 |  |  |  |  |  |
|  | Vomiting | 1 | 1 | 0 | E | 0 | 0 | 0 | 0 | 0 | 0 | 0 | 0 |  |  |  |  |  |
|  | Respiratory distress | 3 | 3 | 0 | E | 0 | 0 | 0 | 0 | 0 | 0 | 0 | 0 |  |  |  |  |  |
|  | Total score | **9** | **11** | **2** | **13** | **0** | **0** | **0** | **0** | **0** | **0** | **0** | **0** | **13** | **13** | **13** | **13** | **13** |
| Total cumulative score | | **27** | **28** | **13** | **31** | **6** | **6** | **6** | **6** | **0** | **3** | **0** | **0** | **53** | **38** | **42** | **50** | **39** |

Supplemental Figure 1: (A) Flow plots for unstained and single-stain (SS) controls. (B) Overall gating strategy for bronchioalveolar lavage fluid (BALF) and peripheral blood mononuclear cells (PBMC).


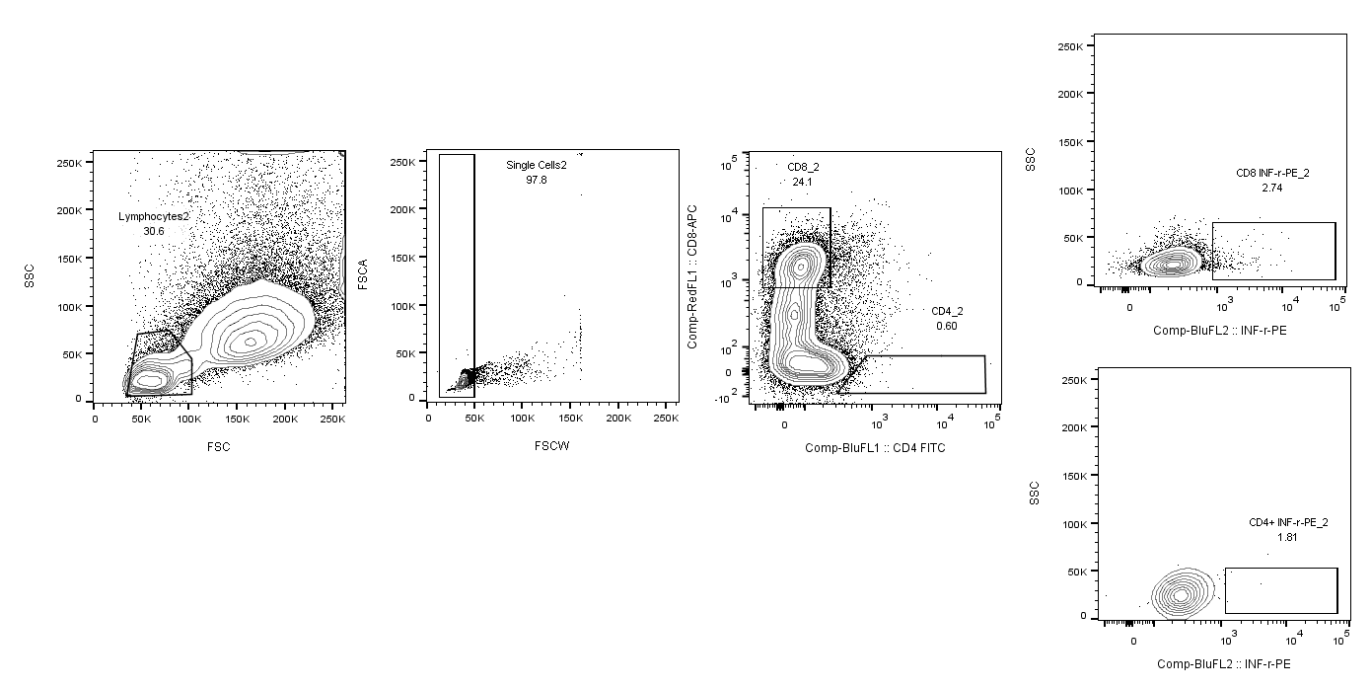

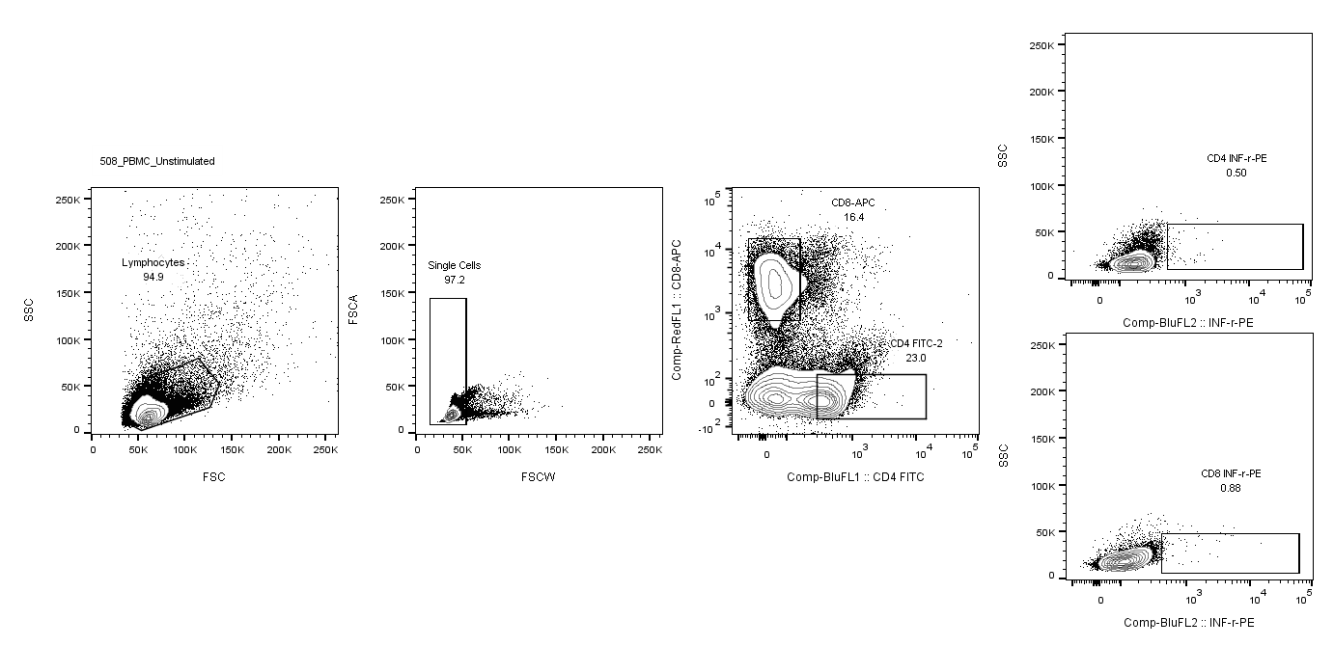


**A.**

**B.**

**Bronchioalveolar Lavage Fluid (BALF)_508**

**PBMC_508**


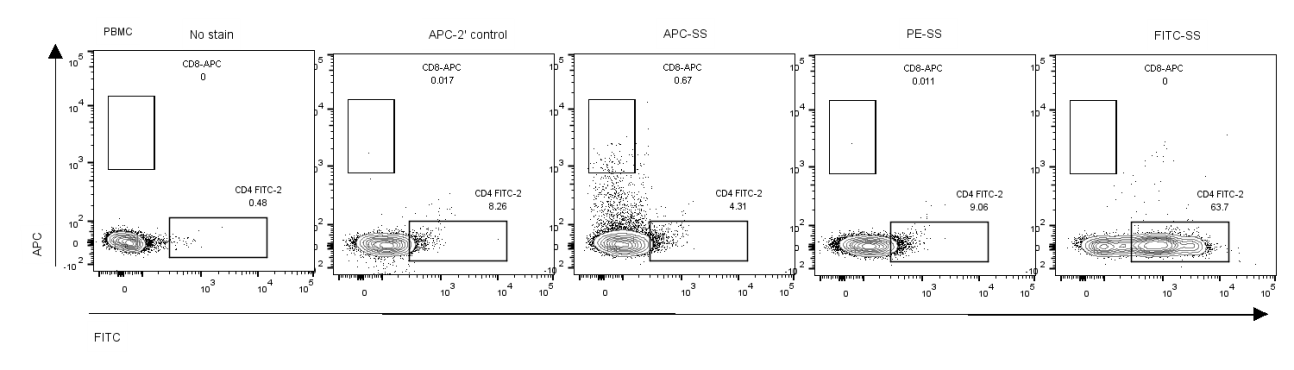

Supplement: Supplementary Information [file srep44727-s1.doc]
